# SIRT6/HIF-1α axis promotes papillary thyroid cancer progression by inducing epithelial–mesenchymal transition

**DOI:** 10.1186/s12935-019-0730-4

**Published:** 2019-01-16

**Authors:** Zhou Yang, Weiping Yu, Renhong Huang, Min Ye, Zhijun Min

**Affiliations:** Department of General Surgery, Shanghai Pudong Hospital, Fudan University Pudong Medical Center, 2800 Gongwei Road, Huinan Town, Pudong, Shanghai, 201399 China

**Keywords:** Sirtuin 6, Hypoxia inducible factor-1α, Papillary thyroid cancer, Epithelial–mesenchymal transition, Sirtuins

## Abstract

**Background:**

In our previous study, we demonstrated that Sirtuin 6 (SIRT6) is upregulated and associated with papillary thyroid cancer (PTC) progression (Qu et al. in Int J Oncol 50(5):1683–92, 2017). This study examined whether SIRT6 promotes epithelial–mesenchymal transition (EMT) of papillary thyroid cancer through hypoxia inducible factor-1α (HIF-1α).

**Methods:**

SIRT6-upregulated TPC-1 and B-CPAP cells were generated by lentivirus. Western blotting, RT-qPCR, immunofluorescence was performed to detect the following EMT associated markers: E-cadherin, Vimentin, Snail, and TWIST. Cell proliferation was detected by CCK8, and cell invasion and migration were detected by transwell and wound healing assays, respectively. HIF-1α expression was further detected by western blotting in both normoxia and hypoxia conditions. A HIF-1α inhibitor was then used to block HIF-1α expression in SIRT6-upregulated PTC cells. The same parameters were then assessed and compared with control HIF-1α cells.

**Results:**

E-cadherin was significantly decreased, whereas Vimentin, Snail, and TWIST were increased in SIRT6-upregulated PTC cells. Additionally, SIRT6 promoted the invasion and migration of PTC cells. We found that SIRT6 enhanced HIF-1α stability and synthesis and prolonged the protein half-life. The changes in the EMT associated markers and in the invasion and migration ability were rescued after inhibition of HIF-1α expression. Furthermore, we found that SIRT6 increased PTC resistance to HIF-1α inhibitor-mediated proliferation changes.

**Conclusion:**

These results confirm that the SIRT6/HIF-1α axis promotes papillary thyroid cancer progression by inducing EMT.

## Background

Thyroid cancer is the most common endocrine malignancy and accounts for 1% of cancers. Papillary thyroid cancer (PTC) is the most common pathological type of thyroid cancer. PTC originates from follicular epithelial cells and represents more than 80% of thyroid cancer [1]. In the past 10 years, the early detection of PTC has improved the patient survival rate, but the overall survival rate of thyroid cancer in nearly 10% patients has not been significantly improved [2]. Therefore, identifying more effective gene targets is critical for thyroid cancer treatment.

The SIRT6 gene is located at chromosome 19p13.3 and contains 8 exons. The encoded protein has a total length of 355 amino acids. SIRT6 protein is a member of the Sirtuins, which is a class of NAD^+^-dependent protein deacetylases involved in stress resistance and metabolic homeostasis [3]. SIRT6 also plays roles in various tumors as both an oncogene and tumor suppressor gene. A previous study in osteosarcoma reported that SIRT6 regulates the migration and invasion of tumors through the ERK1/2/MMP9 pathway [4]. A similar discovery in small cell lung cancer found that upregulation of SIRT6 promotes the invasion of cancer through the ERK1/2/MMP9 pathway [5]. However, in ovarian cancer SIRT6 inhibits tumor proliferation through downregulation of Notch 3 [6]. SIRT6 also suppresses pancreatic cancer progression through control of Lin28b [7].

Our previous study demonstrated that SIRT6 upregulation was associated with poor relapse-free survival (RFS) in PTC patients and enhanced PTC cell migration and invasion in vitro [8]. Epithelial–mesenchymal transition (EMT) is one of the major way tumor cells acquire invasion and migration ability. EMT refers to the biological process of epithelial cells converting into mesenchymal cells. This process is accompanied by decreased E-cadherin and concurrent increases in Vimentin, N-cadherin and the transcription regulators TWIST and Snail [9]. EMT regulation involves a complex network of factors including multiple signaling pathways such as TGF-beta family, Wnts, Notch, EGF, HGF, FGF and HIF. Several studies have explored the relationship between the Sirtuin family and EMT. In both lung cancer and breast cancer SIRT1 promotes EMT and tumor progression [10, 11]. In prostate cancer, SIRT6 can induce EMT and enhance tumor invasion [12]. In colon cancer, SIRT6 promotes EMT through two different ways, one is as a reader of Snail, and other way was the suppression of TET1 transcription. Thus, we hypothesized SIRT6 could induce EMT in PTC.

In this study, we examined the relationship between SIRT6 and EMT in papillary thyroid cancer.

## Methods

### Cell lines and cell culture

Two human PTC cell lines (TPC-1 and B-CPAP) were purchased from the University of Colorado Cancer Center Cell Bank. All cells were cultured in RPMI 1640 medium supplemented with 10% FBS (Invitrogen, Carlsbad, CA, USA) at 37 °C in a 5% CO_2_ atmosphere.

### Generation of SIRT6 stably upregulated cell lines

The cDNA of human SIRT6 was purchased from Origene (RC202833, Rockville, MD, USA) and cloned into the pCDH-CMV-MCS-EF1-Puro lentiviral vector to construct the pCDH-SIRT6 overexpression plasmid. In accordance with the instructions of the product manual, Lipofectamine 3000 (Invitrogen, Inc.) was used to co-transfect the target plasmid or the empty vector, psPAX2, PMG.2G into the HEK293T tool cells to obtain a SIRT6 overexpressed lentivirus or negative control lentivirus. Then, the lentivirus (multiplicity of infection, MOI = 25) was used to infect TPC-1 and B-CPAP cell lines. The SIRT6-upregulated cell lines TPC1-SIRT6 and BCPAP-SIRT6 and empty vector control cell lines TPC1-NC and BCPAP-NC were screened by puromycin (5 μg/mL, 72 h). The overexpression of SIRT6 was confirmed by western blotting.

### Western blot analysis

The total cellular proteins from each group were extracted using RIPA lysis buffer with 1% phenylmethanesulfonyl fluoride (PMSF). Then, equal amounts (20 μg) of protein determined by BCA protein assay kit (Thermo Fisher Scientific, USA) were separated using 10% SDS-PAGE gels. The proteins were then transferred to PVDF membranes (0.45 mm, Solarbio, China). The membranes were blocked with 5% nonfat milk for 1 h at room temperature and then incubated with primary antibodies at 4 °C for 12 h. The following antibodies were tested: SIRT6, Snail1, TWIST1 rabbit polyclonal antibodies, E-cadherin and Vimentin mouse monoclonal antibodies (1:1000, Proteintech, USA), and HIF-1α mouse monoclonal antibody (1:500, Novus Biologicals, USA). α-tubulin or β-actin rabbit polyclonal antibodies (1:4000, Proteintech, USA) were used as loading controls and normalization. The secondary antibodies were anti-mouse or anti-rabbit antibodies and were conjugated to horseradish peroxidase (HRP) (1:4000, Proteintech, USA). The antibodies were used at a 1:4000 dilution and were incubated for approximately 1 h at room temperature. The bands were visualized with ECL reagents (Thermo Fisher Scientific, USA) and developed by Omega Lum G (Aplegen, USA).

### RNA extraction, reverse transcription and quantitative PCR

Total RNA was extracted by Trizol Regent (Invitrogen) from PTC cells. cDNA was obtained from total RNA with PrimeScript™ RT reagent kit (Takara Bio, Inc., Otsu, Japan). The SIRT6 expression was assessed by Real-time quantitative PCR, which was carried out in triplicate by a SYBR Premix Ex Taq™ kit (Takara Bio) and ABI 7900HT Real-Time PCR system (Applied Biosystems Life Technologies, Foster City, CA, USA). The primers used are shown in Table 1. The comparative cycle threshold values (2^−∆∆Ct^) were adopted to analyze the final results.

**Table 1:** The primers of RT-qPCR

Gene	Forward primer	Reverse primer
SIRT6	GCACCGTGGCTAAGGCAAGG	GTGATGGACAGGTCGGCGTTC
Actin	GGGACCTGACTGACTACCTC	TCATACTCCTGCTTGCTGAT
HIF-1α	ACGTTCCTTCGATCAGTTGTCACC	GGCAGTGGTAGTGGTGGCATTAG
E-Cadherin	AGTCACTGACACCAACGATAAT	ATCGTTGTTCACTGGATTTGTG
Vimentin	AGTCCACTGAGTACCGGAGAC	CATTTCACGCATCTGGCGTTC
Snail1	AAGGATCTCCAGGCTCGAAAG	GCTTCGGATGTGCATCTTGA
TWIST	GTACATCGACTTCCTCTACCAG	CATCCTCCAGACCGAGAAG
Slug	CTGTGACAAGGAATATGTGAGC	CTAATGTGTCCTTGAAGCAACC
ZEB1	CAGGCAAAGTAAATATCCCTGC	GGTAAAACTGGGGAGTTAGTCA

### Immunofluorescence (IF)

Coverslips were laid flat on the bottom of a 6-well plate after cleaning, disinfection, and 24 h ultraviolet irradiation. Then, the cells were seeded on the coverslips at a density of 1 × 10^6^ in each well, cultured in an incubator. The coverslips were rinsed with PBS for 5 min 3 times and fixed with 4% paraformaldehyde for 15 min, followed by permeabilization of the cells in 0.3% TritonX-100/PBS for another 20 min. Next, the coverslips were rinsed with PBS again for 5 min 3 times and blocked by incubating cells in 5% BSA for 60 min. Then, the cells were stained with antibodies described in Western blotting method at 1:100 dilution, followed by a 12-h incubation at 4 °C. After washing the uncombined antibody with PBS for 5 min 3 times, the cells were incubated with Alexa 488-coupled Goat Anti-Rabbit IgG secondary antibody or Alexa 594-coupled Goat Anti-Mouse IgG secondary antibody (1:1000, Cell Signaling Technology, USA) for 1 h at room temperature in the darkness. Finally, DAPI was used as a counterstain to label the nuclei. The stained cells were then acquired and photographed with a fluorescent microscope.

### Cell migration and invasion assays

Cell migration and invasion were analyzed by transwell plates (24-well insert, 8 μm pore size; BD Biosciences, Bedford, MA, USA). The filters (Corning, USA) were coated with (invasion) or without (migration) 55 μl Matrigel (1:8 dilution; BD Biosciences). For the migration assays, 2 × 10^4^ cells were suspended in 100 μl serum-free medium and seeded into the Matrigel-uncoated upper chambers. Then, 600 μl of medium containing 10% FBS was added to the lower chamber as a chemoattractant. After incubation at 37 °C for 24 h, the membranes were fixed with 4% formaldehyde for 30 min and stained with 0.1% crystal violet at room temperature for 30 min. For invasion assays, 4 × 10^4^ cells suspended in 100 μl serum-free medium were seeded into the Matrigel-coated upper chambers. The rest of the protocol was identical to that described above. The cells were counted and photographed under an inverted microscope over 5 different fields of each triplicate filter.

### Wound healing assay

For this assay, 5 × 10^5^ cells were seeded into 6-well plates and cultured at 37 °C for 24 h. We used a 200 μl sterile micro-pipette tip to scratch the confluent monolayers in a straight line when cells were 80–90% confluent. Then, we washed floating cells with PBS three times and continued to culture the cells after changing the complete medium to serum-free medium. Images of the same wound position were taken after at 0 h and 24 h under a microscope. The migration results were tested by ImageJ software.

### Cell proliferation assay

For this assay, 2 × 10^3^ cells were seeded into 96-well plates and incubated for the following times: 0 h, 24 h, 48 h and 72 h. Before determination, 10 µl of Cell-Counting Kit-8 (CCK-8; Dojindo, JAP) solution was added to each well of the plate, and the incubation was continued for 2 h. Finally, we measured the absorbance of each well at a 450 nm wavelength.

### Drug treatment

HIF-1α inhibitor (YC-1) was purchased from Selleck (Huston, USA), and the working concentration was 10 μM for 24 h as recommended. Hypoxia induced drug cobalt chloride (CoCl_2_) was purchased from Sigma-Aldrich (CA, USA), and the working concentration was 200 μM for 24 h, as described in a previous study [13]. The effects of both compound treatments were confirmed by western blotting.

### Statistical analysis

SPSS software (version 19.0, IBM Corp., Armonk, NY, USA) was used for statistical analysis of all the experimental data. GraphPad Prism (version 7, GraphPad Software, La Jolla, CA, USA) was used to determine the statistical results. All data are expressed as the mean + standard deviation (mean + SD). The statistical analysis of the data from 2 groups was performed using a t-test. The comparisons of multiple groups were performed by one-way ANOVA and then an LSD-t test. P < 0.05 was considered to be significant.

## Results

### SIRT6 was successfully upregulated in TPC1-SIRT6 and BCPAP-SIRT6

**Fig. 1:**
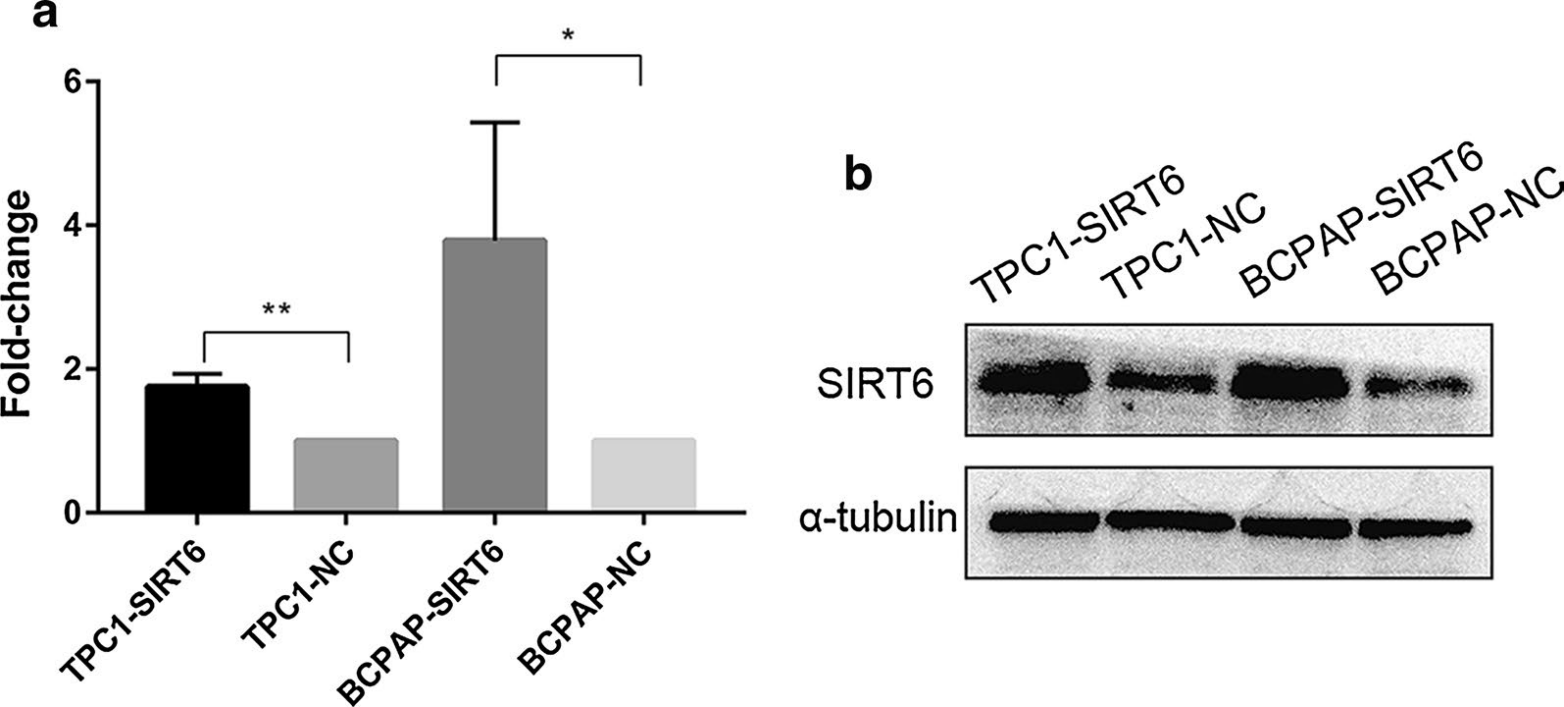
**a** SIRT6 expression confirmed by real-time quantitative PCR. **b** SIRT6 expression confirmed by western blotting. (*p < 0.05, **p < 0.01, ***p < 0.001)

We chose two common papillary thyroid cancer cell lines, namely, TPC-1 and B-CPAP, to overexpressSIRT6. The results of western blotting and Real-time quantitative PCR (Fig. 1) showed that the expression of SIRT6 protein in TPC1-SIRT6 and BCPAP-SIRT6 increased significantly compared with that of the negative control TPC1-NC and BCPAP-NC. These results indicated that we successfully generated stably SIRT6-upregulated PTC cells.

### Upregulation of SIRT6-induced EMT in PTC cells

**Fig. 2:**
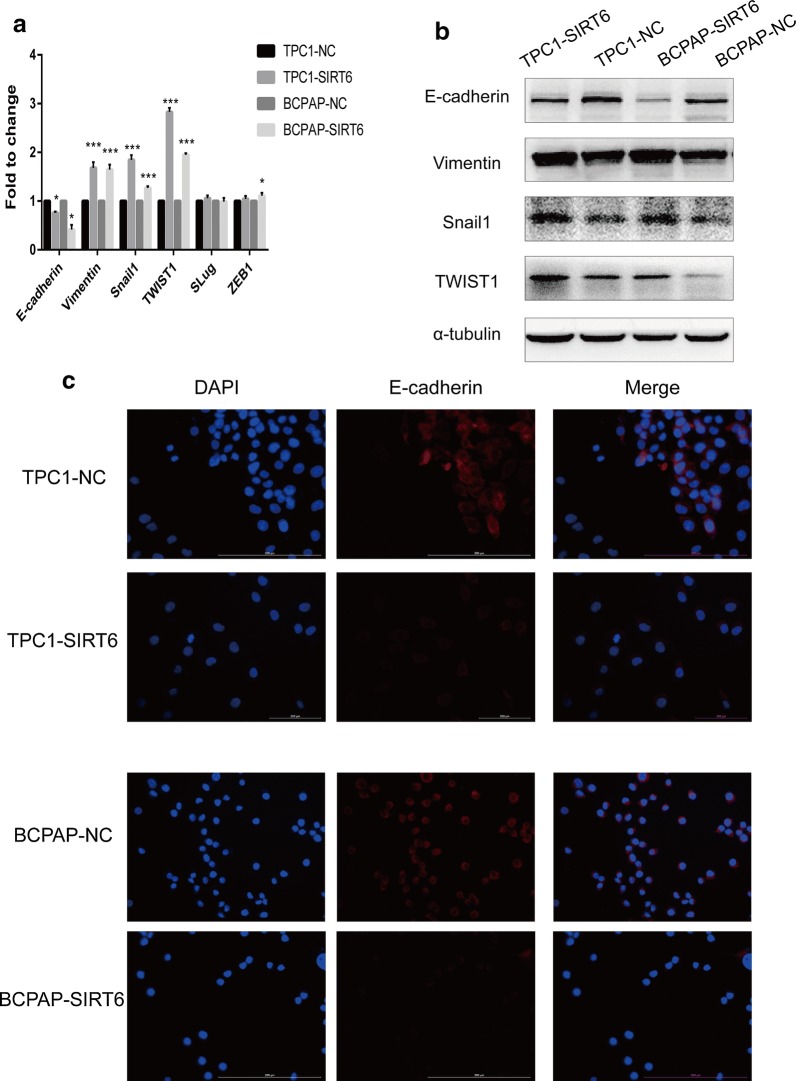
Expression of EMT associated markers. **a** EMT associated markers expression analyzed by RT-qPCR. **b** EMT associated markers expression detected by Western Blotting. **c** Expression and location of E-cadherin in PTC cells analyzed by IF. (*p < 0.05, **p < 0.01, ***p < 0.001)

RT-qPCR was performed to detect main EMT associated markers mRNA expression (Fig. 2a). E-cadherin showed significantly decrease in SIRT6 upregulated PTC cells; meanwhile, Vimentin, Snail1, TWIST showed significantly increase. Whereas there was no obvious change in Slug and ZEB1. Furthermore, Western blotting was performed to detect the protein expression of E-cadherin, Vimentin, Snail, and TWIST (Fig. 2b). The changing trend tallied the result of RT-qPCR. Finally, IF was used to analysis E-cadherin expression and location (Fig. 2c). E-cadherin showed the same significantly decrease in SIRT6 upregulated cells. Meanwhile, E-cadherin located in cell membrane in all PTC cells.

### Overexpression of SIRT6 increased PTC cells migration and invasion in vitro

**Fig. 3:**
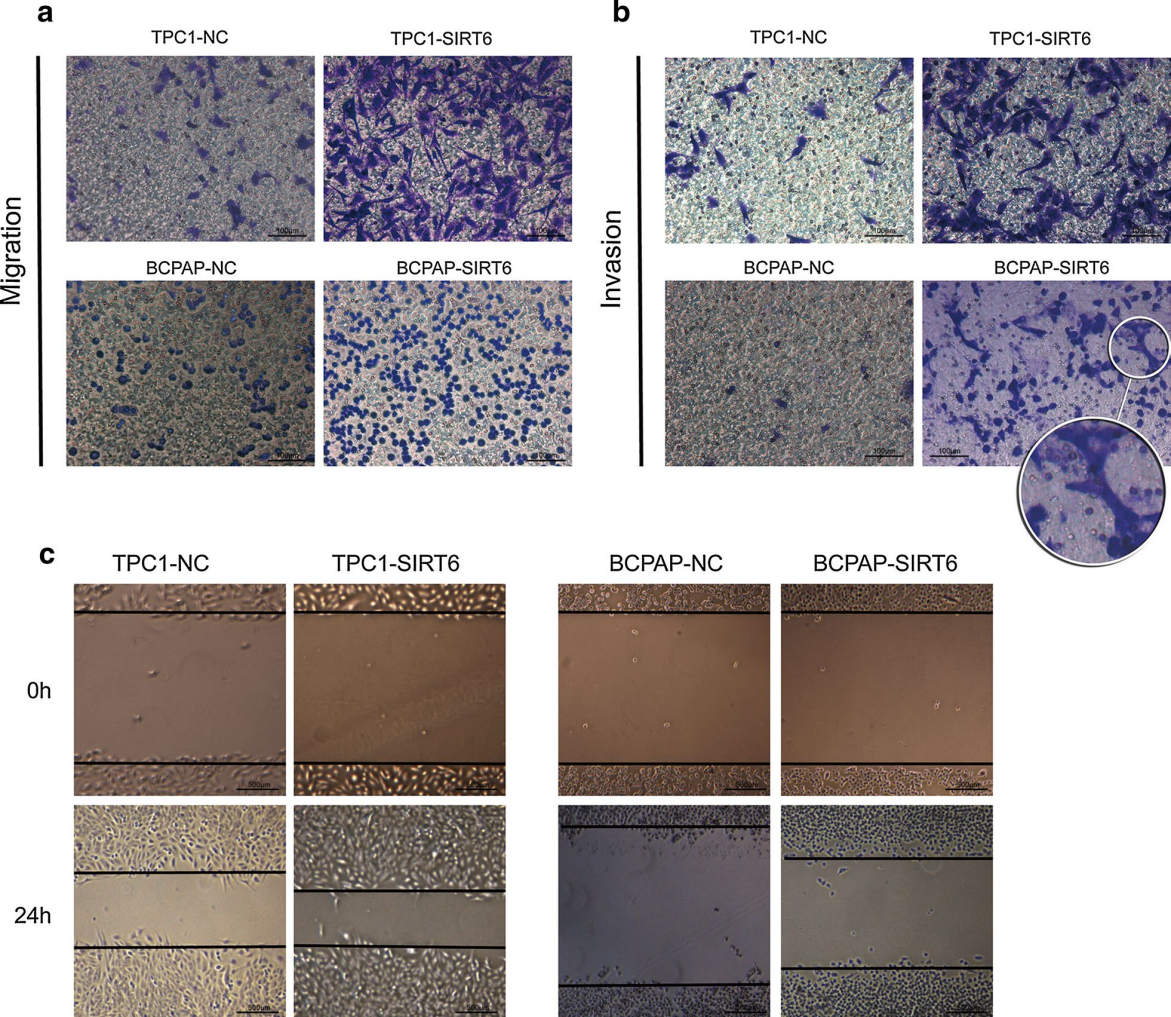
Upregulating SIRT6 increased PTC cell migration and invasion. **a** TPC1-SIRT6 and BCPAP-SIRT6 showed significant increases in migration ability. **b** TPC1-SIRT6 and BCPAP-SIRT6 showed a significant increase in invasion ability. **c** TPC1-SIRT6 and BCPAP-SIRT6 showed a smaller wound area after 24 h

The transwell and wound healing assays were used to evaluate the migration and invasion of PTC cells. There were more TPC1-SIRT6 and BCPAP-SIRT6 cells than TPC1-NC and BCPAP-NC cells in both the migration and invasion assays (Fig. 3a, b). In the invasion assay, we also found that a fraction of the BCPAP-SIRT6 cells changed shape from spherical to fusiform. However, we did not find this morphological difference in BCPAP-NC cells in the invasion assay or in the BCPAP-SIRT6 and BCPAP-NC cells in the migration assay. In the wound healing assay, TPC1-SIRT6 and BCPAP-SIRT6 cells showed a smaller wound area after 24 h (Fig. 3c). Collectively, these results indicated that SIRT6 promotes both PTC cell invasion and migration.

### SIRT6 increased HIF-1α expression by enhancing its stability and synthesis and by prolonging its half-life

**Fig. 4:**
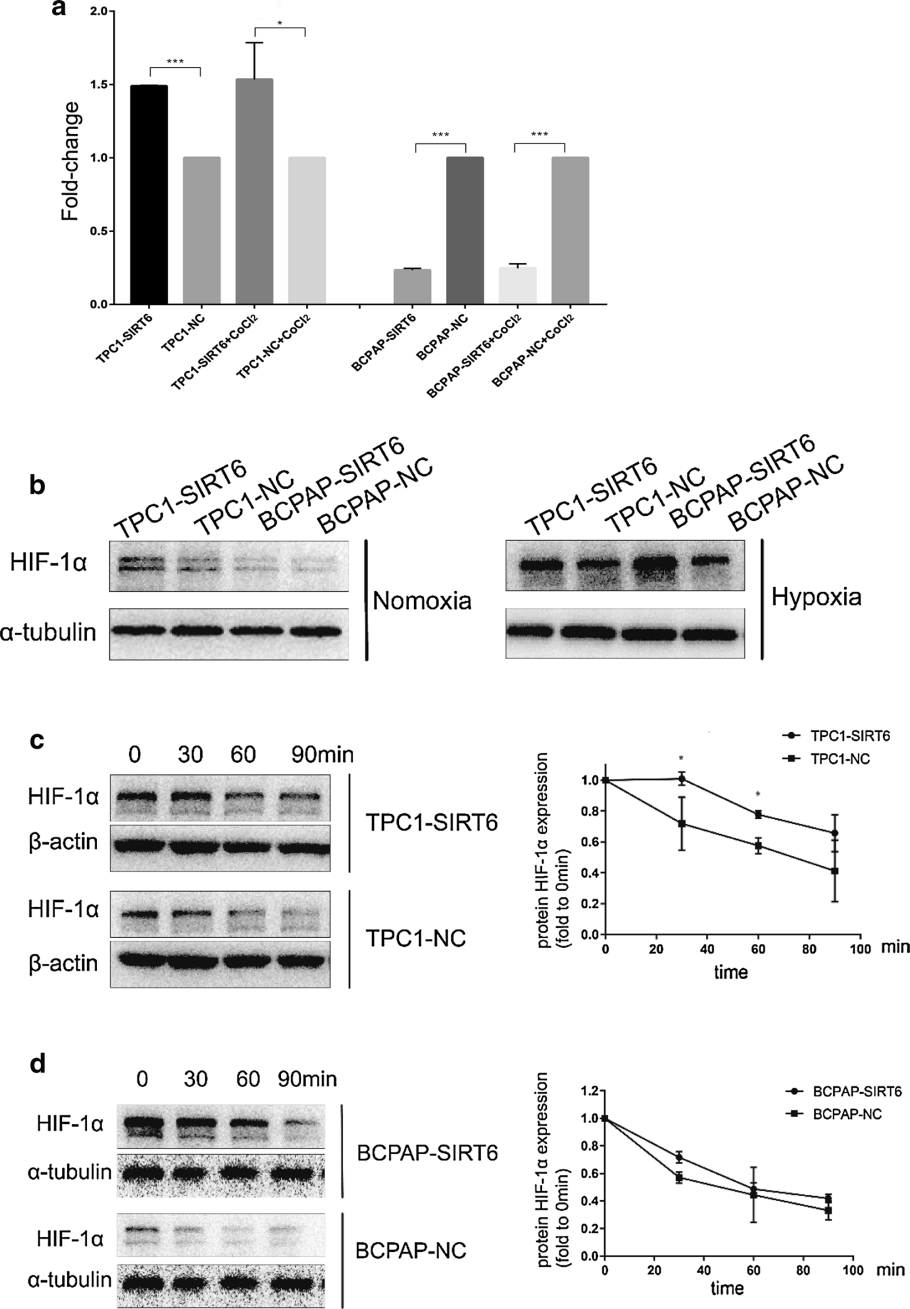
**a** mRNA expression of HIF-1α fold to negative control under normoxia and hypoxia. The comparative cycle threshold values (2^−∆∆Ct^) and t-test were adopted to analyze the final results. **b** Protein expression of HIF-1α in each group under normoxia and hypoxia. **c** HIF-1α expression in TPC1-SIRT6 and TPC1-NC cells after withdrawing CoCl_2_ for 0–90 min. **d** HIF-1α expression in BCPAP-SIRT6 and BCPAP-NC cells after withdrawing CoCl_2_ for 0–90 min. (*p < 0.05, **p < 0.01, ***p < 0.001)

**Fig. 5:**
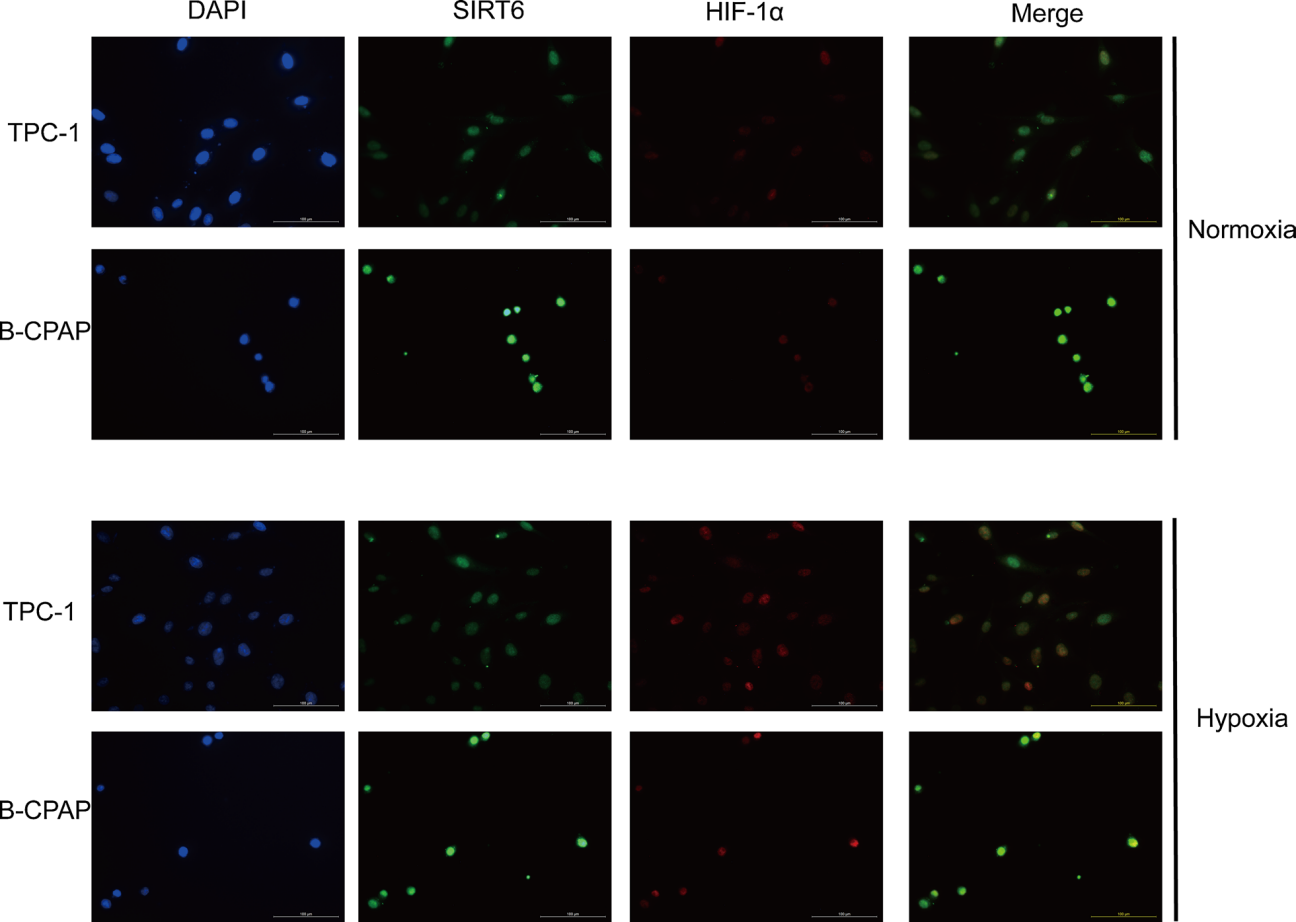
Double-immunofluorescent of HIF-1α and SIRT6 under both normoxia and hypoxia

The normoxia group was incubated as described. The hypoxia group was incubated with 200 μM CoCl_2_ for 24 h. Real-time quantitative PCR was performed to detect mRNA expression of HIF-1α in both normoxia and hypoxia (Fig. 4a). The expression of HIF-1α in different groups was normalized by negative control. In both normoxia and hypoxia group, the expression of HIF-1α in TPC1-SIRT6 was significantly upregulated compared with TPC1-NC; However, HIF-1α of BCPAP-SIRT6 was down-regulated compared with BCPAP-NC. And we also found none significant differences in HIF-1α expression fold change of TPC1-SIRT6/TPC1-NC nor BCPAP-SIRT6/BCPAP-NC between normoxia and hypoxia groups. We then further detected protein expression of HIF-1α in normoxia and hypoxia by western blotting (Fig. 4b). In normoxia group, the expression of HIF-1α in TPC1-SIRT6 was significantly upregulated compared with TPC1-NC; Whereas HIF-1α in both BCPAP-SIRT6 and BCPAP-NC cells were weakly detected. In the hypoxia group, HIF-1α was obviously upregulated in BCPAP-SIRT6 cells compared with BCPAP-NC cells. We also obtained a similar but less obvious result in TPC1-SIRT6 cells. We then removed CoCl_2_ in the hypoxia group and extracted total protein at different times (0, 30, 60, 90 min) after the withdraw to perform western blotting (Fig. 4c, d). The HIF-1α expression of each group at 30, 60, 90 min was normalized by the expression level at 0 min. The HIF-1α level in TPC1-SIRT6 cells degenerated significantly slower than that of TPC-NC cells (p < 0.05). We also found slower degeneration of HIF-1α in BCPAP-SIRT6 cells compared with that of BCPAP-NC cells, but no statistically difference was observed. Furthermore, double-immunofluorescent staining of TPC-1 and B-CPAP cells was performed (Fig. 5). SIRT6 and HIF-1α both located in nucleus, and obvious co-localization was infirmed under both normoxia and hypoxia.

### Inhibition of HIF-1α rescued SIRT6-upregulated PTC cells from EMT

**Fig. 6:**
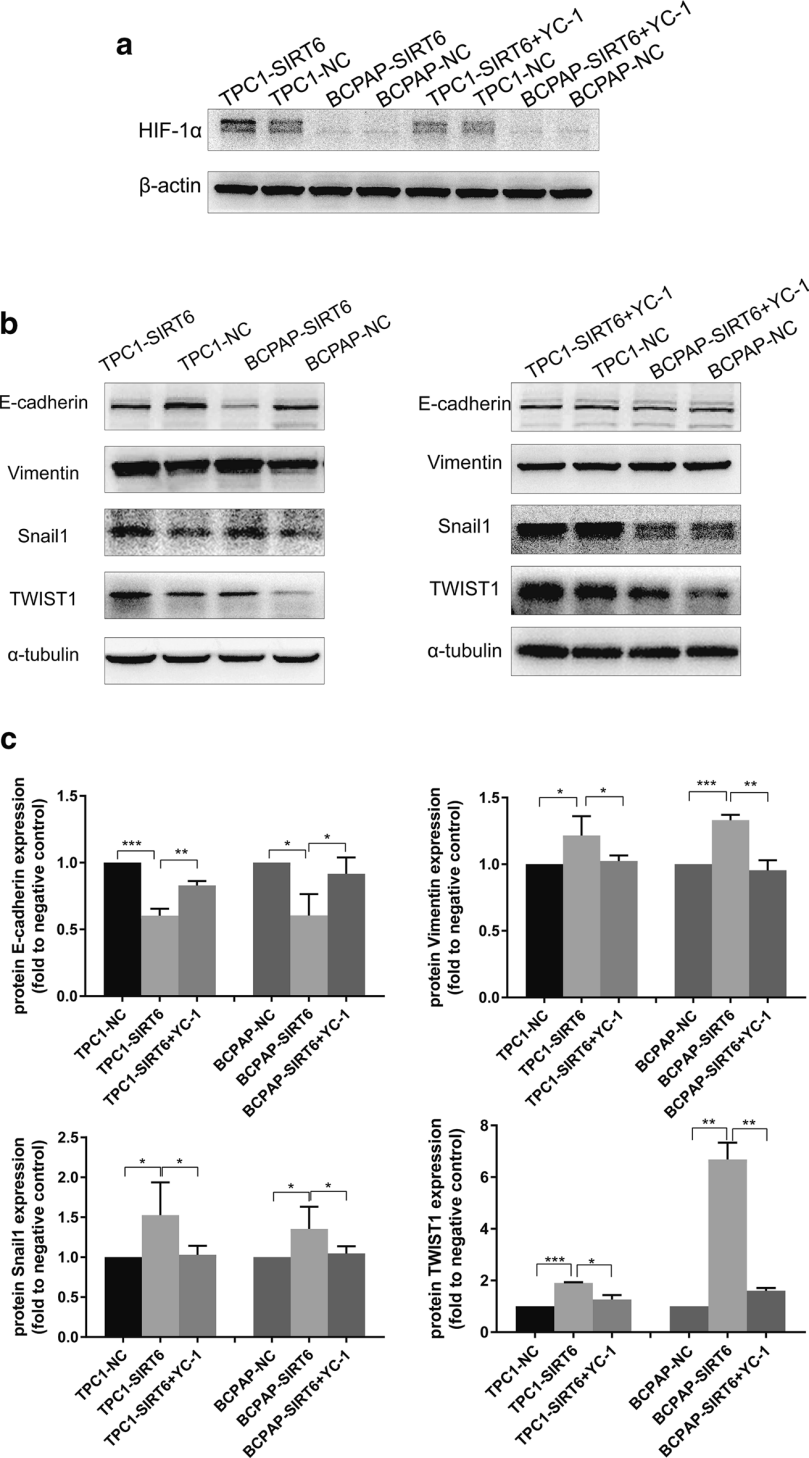
**a** HIF-1α expression detected by western blotting after inhibition of YC-1. **b** EMT-associated markers detected by western blotting (reuse Fig. 2). **c** The intensity of EMT associated markers normalized by negative control. (*p < 0.05, **p < 0.01, ***p < 0.001)

YC-1 is a HIF-1α inhibitor that functions at the post-transcriptional level [14]. Therefore, 10 μM YC-1 was added to the medium of TPC1-SIRT6 and BCPAP-SIRT6 and then incubated for 24 h. HIF-1α expression was significantly inhibited by YC-1 (Fig. 6a). We then further detected EMT associated markers of TPC1-SIRT6 and BCPAP-SIRT6 after inhibiting HIF-1α. The decrease in E-cadherin and increase in Vimentin, Snail, and TWIST in TPC1-SIRT6 and BCPAP-SIRT6 were rescued at different levels that were all statistically significant (Fig. 6b, c).

### Upregulation of migration and invasion in SIRT6-overexpressed PTC cells rescued after inhibiting HIF-1α

**Fig. 7:**
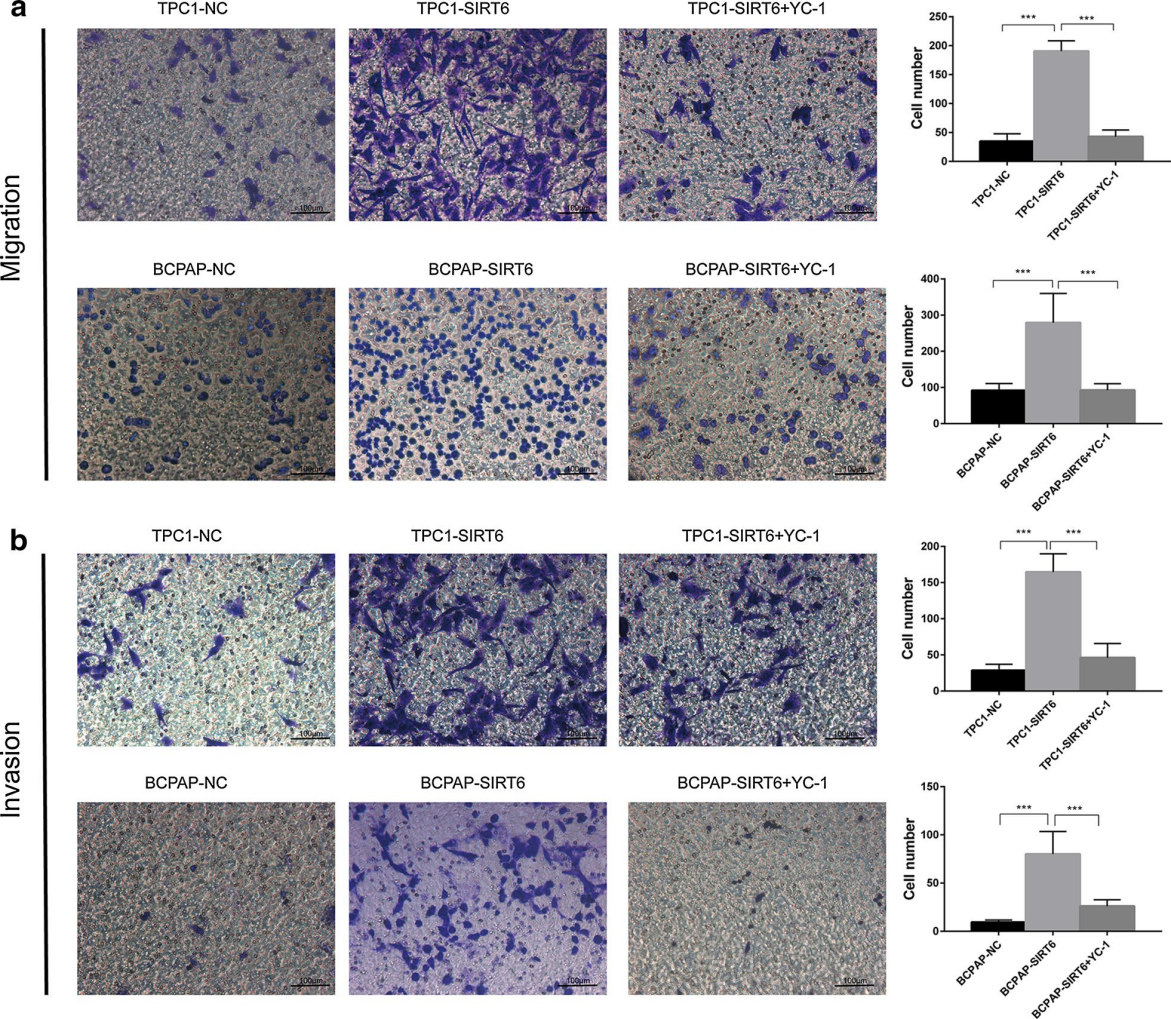
YC-1 pretreatment inhibited cell invasion and migration. (Reuse Fig. 3a, b). **a** The migration assay showed that YC-1 inhibited the increased migration ability of TPC1-SIRT6 and BCPAP-SIRT6 cells. **b** The invasion assay showed that YC-1 inhibited the increased invasive ability of TPC1-SIRT6 and BCPAP-SIRT6 cells. (*p < 0.05, **p < 0.01, ***p < 0.001)

**Fig. 8:**
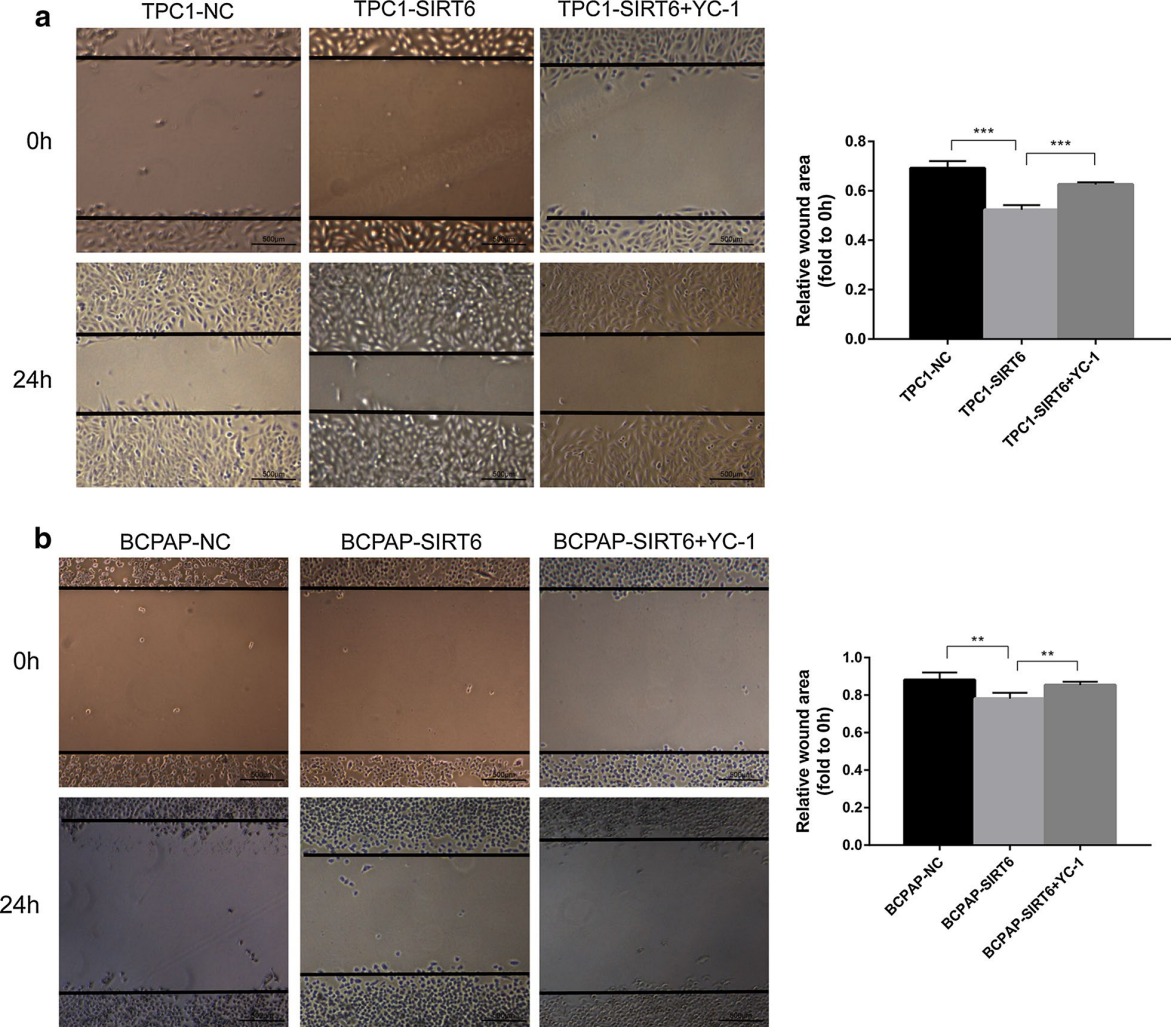
**a** YC-1 counteracted the reduction of the wound area in TPC1-SIRT6 cells. **b** YC-1 counteracted the reduction of the wound area in BCPAP-SIRT6 cells. (Reuse Fig. 3c, *p < 0.05, **p < 0.01, ***p < 0.001)

After pretreatment with 10 μM YC-1 for 24 h, TPC1-SIRT6 and BCPAP-SIRT6 cells were tested in the cell migration and invasion assays described above (Fig. 7). The data showed that both the invasion and migration abilities of TPC1-SIRT6 and BCPAP-SIRT6 cells decreased. Interestingly, the change from a spherical to fusiform shape in the BCPAP-SIRT6 cells in the invasion assay described above also disappeared. In the wound healing assay, the wound area of TPC1-SIRT6 and BCPAP-SIRT6 treated with YC-1 also recovered (Fig. 8).

### SIRT6-upregulated cells showed stronger resistance to a HIF-1α inhibitor in cell proliferation

**Fig. 9:**
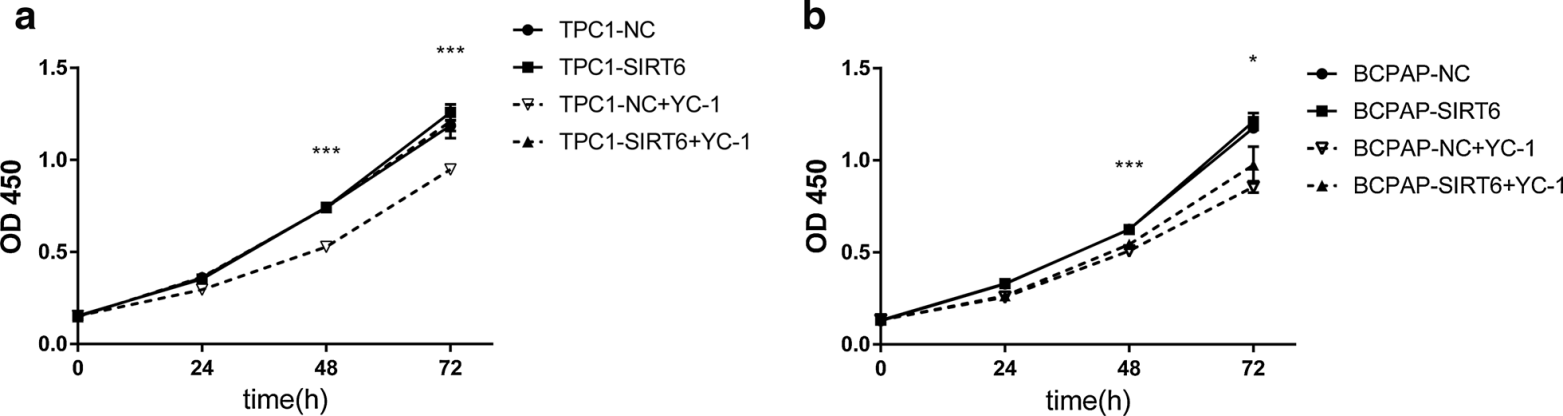
Cell proliferation of each group detected by CCK8. **a** TPC1-SIRT6+YC-1 group showed a stronger proliferation compared with TPC1-NC+YC-1 group. **b** BCPAP-SIRT6+YC-1 group showed a stronger proliferation compared with BCPAP-NC+YC-1 group (*p < 0.05, **p < 0.01, ***p < 0.001)

CCK8 was used to detect cell proliferation. We found no changes in cell proliferation between TPC1-SIRT6 or BCPAP-SIRT6 and their negative controls. We then further used the HIF-1α inhibitor YC-1 to treat each group for 24 h. Interestingly, we found that YC-1 significantly inhibited TPC1-NC proliferation. However, there was almost no effect on TPC1-SIRT6 cells (Fig. 9a). A similar diminished resistance was also observed in BCPAP-SIT6 cells (Fig. 9b).

## Discussion

SIRT6 has been reported to have multiple roles in previous studies of different tumors, but there is currently no report on thyroid cancer. Our early work infirmed that SIRT6 promotes papillary thyroid cancer aggression. We also found that SIRT6 enhanced PTC cell migration and invasion in vitro. However, the specific mechanism had not been studied. EMT is well known as one of the most important ways for tumor cells to acquire invasion and migration abilities. Thus, we conducted further research into the relationship between SIRT6 and EMT.

We successfully generated SIRT6-upregulated PTC cells, specifically TPC1-SIRT6, BCPAP-SIRT6 and their negative controls TPC1-NC and BCPAP-NC. Western blotting, RT-qPCR and IF were performed to detect the key EMT associated markers. There was a significant decrease in E-cadherin and increase in Vimentin, Snail, and TWIST in TPC1-SIRT6 and BCPAP-SIRT6 cells. These results indicate that SIRT6 promoted EMT in PTC cells. Transwell and wound healing assays were then used to confirm the cell migration and invasion in vitro. Both TPC1-SIRT6 and BCPAP-SIRT6 showed a stronger invasion and migration ability than did their respective negative controls. It was noteworthy that a subset of BCPAP-SIRT6 which was naturally sphere acquired a fusiform shape during the invasion assay. This translation met the morphologic change of EMT. These data indicated that SIRT6 promoted PTC migration and invasion via inducing EMT.

EMT is regulated by a complex network associated with various molecular and pathways. We then focused our research on HIF-1α, which is one of the key molecular regulators of EMT. HIF-1α is nuclear protein with transcriptional activity and it has a wide spectrum of target genes. Previous studies have reported many genes can induce EMT by interacting with HIF-1α [15, 16]. The Sirtuin family can also interact with HIF in a variety of ways including inhibition of transcription (SIRT1) and regulation of stability (SIRT3, SIRT6, SIRT7) [10, 17, 18]. At mRNA level, we found SIRT6 increased HIF-1α expression in TPC-1, whereas decreased HIF-1α in B-CPAP cell. HIF-1α protein is unstable in normoxia, and the protein is degraded by oxygen-dependent ubiquitin protease soon after synthesis. Thus, it can exist stably only under hypoxia. However, we found TPC1-SIRT6 cells had a more stable overexpression of HIF-1α compared with TPC1-NC in normoxia. HIF-1α in BCPAP-SIRT6 and BCPAP-NC was weakly detected, and we used CoCl_2_ to simulate hypoxia because it prevents HIF-1α from degeneration by replacing the prolyl hydroxylase (PHD) cofactor Fe^2+^ [19]. HIF-1α in BCPAP-SIRT6 cells was significantly upregulated compared with BCPAP-NC in hypoxia, yet the upregulation of HIF-1α in TPC1-SIRT6 was not obvious. These results indicated that HIF-1α of TPC1-SIRT6 is already stable in normoxia and that SIRT6 also increases the HIF-1α expression in both TPC1-SIRT6 and BCPAP-SIRT6 cells in hypoxia. CoCl_2_ was then removed, and Western blotting was used to detect HIF-1α expression at various time points after the withdrawal. We found a significantly slower degeneration of HIF-1α in TPC1-SIRT6 and a similar but less obvious result in BCPAP-SIRT6 cells. Furthermore, co-localization of SIRT6 and HIF-1α in PTC cells was also confirmed in both normoxia and hypoxia. These findings strengthened our conclusion that SIRT6 increases HIF-1α stability, prolongs its half-life (especially in TPC-1) and increases its synthesis (especially in B-CPAP under hypoxia) in PTC cells.

To further investigate SIRT6/HIF-1α axis in EMT, YC-1 was used to inhibit HIF-1α expression in TPC1-SIRT6 and BCPAP-SIRT6 cells. YC-1 degrades the C-terminal end of HIF-1α to inhibit its activity at a post -transcriptional level [14]. We found a similar level but an opposite effect compared with that for SIRT6. The treatment with 10 μM YC-1 for 24 h succeeded in inhibiting HIF-1α expression. Notably, all the changes in the EMT associated markers were rescued at different levels. The increase in the invasion and migration ability recovered, and the morphological changes in BCPAP-SIRT6 also disappeared. Additionally, we found TPC1-SIRT6 and BCPAP-SIRT6 cells had a stronger resistance to YC-1 in cell proliferation compared with negative controls.

HIF-1α is reported to regulate the expression of Snail and TWIST by binding directly to the hypoxia-response element (HRE) in their promoters [20, 21]. Snail and TWIST also work as key EMT-inducing transcription factors that regulate E-cadherin and Vimentin expression [22, 23]. SIRT6 upregulated HIF-1α expression in both normoxia and hypoxia. Then, stably upregulated HIF-1α binds to the HRE of Snail and TWIST to increase their expression. The overexpression of the Snail and TWIST transcription factors then regulates the decrease in E-cadherin and increase in Vimentin. Taken together, these molecular changes induce EMT. EMT promotes PTC cell invasion and migration ability and may result in poor RFS in PTC patients.

There are several differences such as the interaction with HIF-1α and resistance to YC-1 in proliferation between TPC1-SIRT6 and BCPAP-SIRT6 that cannot be well explained. TPC-1 cells harbor the BRAF^V600E^ mutation, whereas the BCPAP cell line has BRAF^V600E^ mutation. The BRAF^V600E^ mutation is also the most regular oncogenic mutation in PTC associated with a poor prognosis. Therefore, we hypothesize that the BRAF^V600E^ mutation associates and interacts with the SIRT6/HIF-1α axis. We will focus on testing this hypothesis in our next study. Taken together, our data suggest that the SIRT6/HIF-1α axis promotes PTC progression by inducing EMT. The SIRT6/HIF-1α axis could be a new therapeutic target and diagnostic marker of papillary thyroid cancer.

## Conclusion

Combined with our previous study, we could safely draw the conclusion that upregulated SIRT6 in PTC inducing epithelial–mesenchymal transition by positive regulation of HIF-1α, thus promotes tumor progression. This is a study infirmed SIRT6/HIF-1α axis in thyroid cancer for the first time.
