# Correction to: SIRT6/HIF-1α axis promotes papillary thyroid cancer progression by inducing epithelial–mesenchymal transition

**DOI:** 10.1186/s12935-023-03015-4

**Published:** 2023-08-22

**Authors:** Zhou Yang, Weiping Yu, Renhong Huang, Min Ye, Zhijun Min

**Affiliations:** Department of General Surgery, Shanghai Pudong Hospital, Fudan University Pudong Medical Center, 2800 Gongwei Road, Huinan Town, 201399 Pudong, Shanghai China

**Correction to:** ***Cancer Cell Int (2019)*** **19:17**

10.1186/s12935-019-0730-4

In the article [1], the authors have found an error of α-tubulin in Fig. 4d. This error was caused by the same label name, and we put a same picture of α-tubulin in both BCPAP-NC and BCPAP-SIRT6 group in Fig. 4d during figure processing. The error doesn’t affect any results and conclusions of this research. The correct Fig. 4d is given in this correction.

**Fig. 4:**
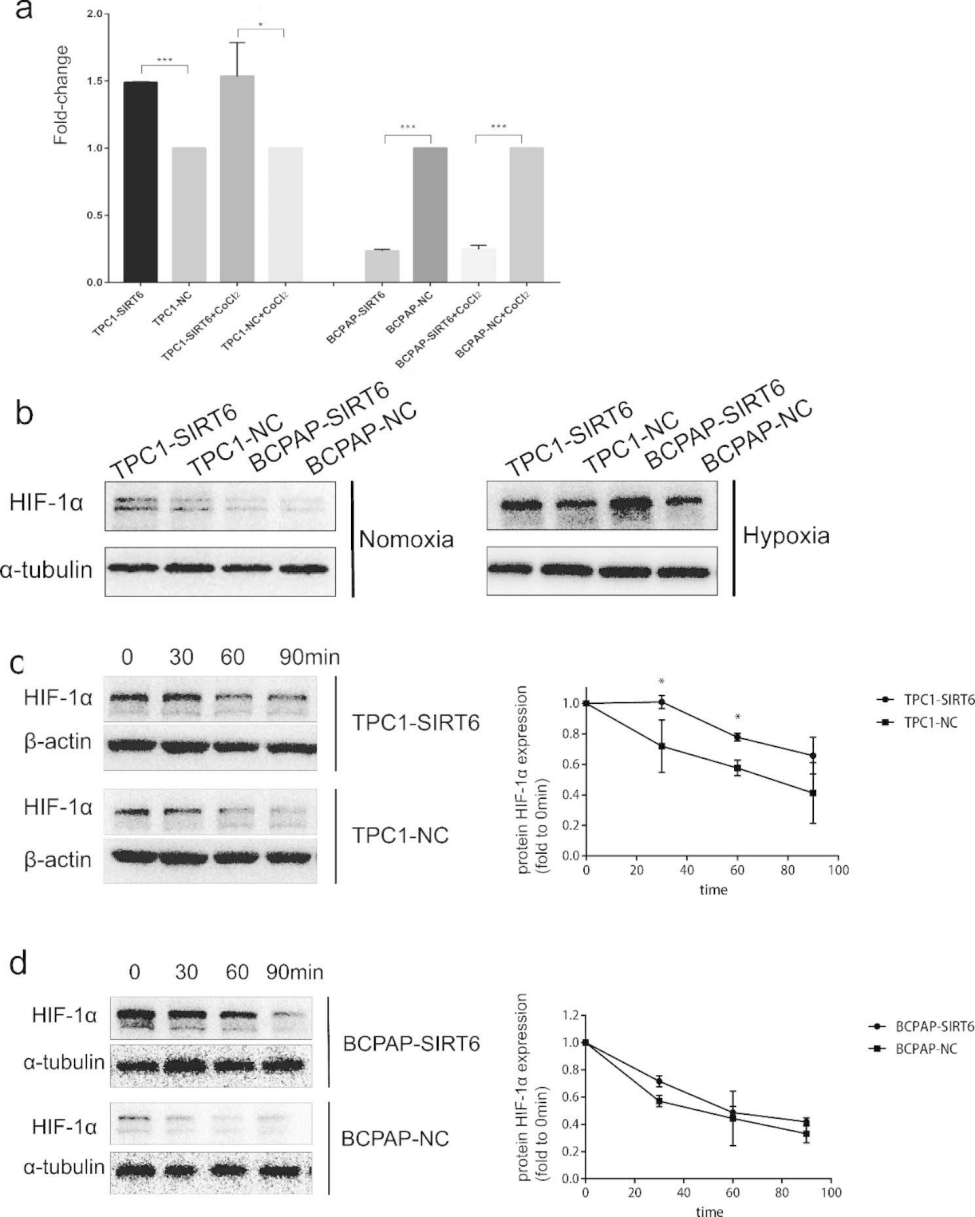
**a** mRNA expression of HIF-1α fold to negative control under normoxia and hypoxia. The comparative cycle threshold values (2−∆∆Ct) and t-test were adopted to analyze the final results. **b** Protein expression of HIF-1α in each group under normoxia and hypoxia. **c** HIF-1α expression in TPC1-SIRT6 and TPC1-NC cells after withdrawing CoCl2 for 0–90 min. **d** HIF-1α expression in BCPAP-SIRT6 and BCPAP-NC cells after withdrawing CoCl2 for 0–90 min. (*p < 0.05, **p < 0.01, ***p < 0.001)
